# The Pathophysiology of Alcohol-Associated Liver Disease: Focusing on Superoxide Dismutase 1 as a Therapeutic Target

**DOI:** 10.3390/biology14101319

**Published:** 2025-09-24

**Authors:** Thiyagarajan Gopal, Arul Daniel John Kathiravan, Alexander V. Kabanov, Carol A. Casey, Viswanathan Saraswathi

**Affiliations:** 1 Centre for Laboratory Animal Technology and Research, Sathyabama Institute of Science and Technology, Chennai 600119, Tamil Nadu, India; thiyagarajan.clatr@sathyabama.ac.in; 2 Department of Internal Medicine, Division of Diabetes, Endocrinology, and Metabolism, University of Nebraska Medical Center, Omaha, NE 68198, USA; ajohnkathiravan@unmc.edu; 3 VA Nebraska-Western Iowa Health Care System, Omaha, NE 68105, USA; ccasey@unmc.edu; 4 Division of Pharmacoengineering and Molecular Pharmaceutics, Eshelman School of Pharmacy, University of North Carolina, Chapel Hill, NC 27599, USA; kabanov@unc.edu; 5 Department of Internal Medicine, Division of Gastroenterology and Hepatology, University of Nebraska Medical Center, Omaha, NE 68198, USA

**Keywords:** alcohol-associated liver disease, SOD1, NanoSOD1, ROS

## Abstract

Alcohol-associated liver disease (ALD) is a major health problem of global importance, caused by chronic alcohol consumption, leading to the accumulation of reactive oxygen species (ROS) and subsequent oxidative stress—a central mechanism in liver injury. Superoxide dismutase 1 (SOD1), a Cu-Zn containing antioxidant enzyme, plays a crucial role in attenuating ALD-induced oxidative stress triggered by ethanol metabolism. However, alcohol exposure, whether chronic, acute or binge, differentially affects SOD1 levels, either diminishing its expression or temporarily compensating for alcohol-induced oxidative damage. Regardless, overexpression of SOD1 reverses early stages of ethanol-induced liver inflammation and injury in animal models, highlighting the protective role of SOD1. Current therapies, including alcohol abstinence, corticosteroids, and pentoxifylline, have limited long-term efficacy. Antioxidant-based treatments, such as N-acetylcysteine (NAC) and S-adenosyl-L-methionine (SAM), have demonstrated moderate benefits. While combination therapies like NAC with prednisolone yield more promising outcomes, these benefits are often limited in duration. The use of natural compounds including nutraceuticals and probiotics provide liver protection by enhancing antioxidant defenses, reducing inflammation, and mitigating alcohol-induced liver damage. In particular, these compounds upregulate antioxidant enzymes like SOD1. Recent research suggests that enhancing the activity of SOD1, particularly through nanoformulated SOD1 (NanoSOD1), which had direct effect on the oxidative stress at the cellular level, could offer a promising therapeutic option for ALD. NanoSOD1 aims to improve the bioavailability and stability of SOD1, offering a targeted approach to reduce oxidative stress and protect against liver damage. The effectiveness of NanoSOD1 to improve antioxidant defenses suggests a valuable therapeutic arsenal in ALD treatment. Taken together, given the limited treatment options for ALD, increasing SOD1 activity is essential for managing the progression of the disease.

## Simple Summary

Alcohol-associated liver disease (ALD) develops from long-term alcohol use, which causes harmful molecules to build up in the liver and trigger damage. An enzyme called Superoxide dismutase 1 (SOD1) helps protect liver cells by reducing this oxidative stress, and boosting its levels has been shown to reverse early liver injury in research studies. Existing treatments like steroids and antioxidants offer only short-term benefits. Many of the alternative therapies are effective in preventing ALD and act, at least in part, by increasing SOD1 levels. A newer approach using NanoSOD1, a specially engineered form of SOD1 enzyme, shows promise for long-term protection. NanoSOD1 is designed to be more stable and better absorbed by the body, allowing it to reach liver cells more effectively. By strengthening the body’s antioxidant defenses, it may offer safer and more targeted therapy for people with alcohol-associated liver damage.

## 1. Introduction

Heavy or prolonged alcohol consumption results in alcohol-associated liver disease (ALD), which has grown to be a serious public health problem [1,2]. According to the World Health Organization (WHO) global status report, about 2.3 billion people currently consume alcohol, and every year, about 3 million deaths are associated with ALD, making it one of the top five causes of global mortality [1,2]. Consumption of alcohol causes a wide spectrum of liver injuries ranging from simple hepatic steatosis (lipid accumulation in the liver) to steatohepatitis (steatosis with inflammation), alcohol-associated hepatitis (AH), fibrosis (inflammation, infection, and severe injury), cirrhosis (severe scarring of tissue), and finally hepatocellular carcinoma (HCC), an advanced stage of liver disease [1,3,4]. Despite the severity and prevalence of ALD, there are currently no US Food and Drug Administration (FDA)-approved acceptable therapeutics exist to reverse the disease progression [5]. Corticosteroids (prednisolone) and pentoxifylline are available for short-term survival in patients with severe alcoholic hepatitis [4,6]. To date, liver transplantation is still the best and most promising treatment option for patients with decompensated liver injury [7,8,9].

Numerous reports have demonstrated that the pathogenesis of ALD is accompanied by oxidative stress and inflammatory injury [10,11,12,13,14]. Chronic and excessive ethanol induces the activation of Cytochrome P450 Family 2 Subfamily E Member 1 (CYP2E1), a key microsomal enzyme responsible for ethanol metabolism, which generates acetaldehyde and superoxide O_2_•^−^ radicals as toxic byproducts. Exacerbated ethanol-induced oxidative stress is the crucial mechanism involved in the pathogenesis of ALD. During ethanol metabolism, an imbalance between the generation of reactive oxygen species (ROS) and antioxidant defense activates oxidative stress and influences the development of ALD. Subsequently, the increased production of ROS triggers the activation of Kupffer cells, leading to the generation of proinflammatory cytokines including interleukins (IL1β and IL6) and tumor necrosis factor-alpha (TNFα) in the liver, thereby promoting inflammation and hepatic injury.

Superoxide dismutase (SOD) is the primary ROS-scavenging enzyme involved in the clearance of O_2_•^−^ generated during ethanol metabolism. Of note, excessive alcohol consumption downregulates the synthesis and enzymatic activity of SOD1 or copper-zinc SOD in the liver. Diminished SOD activity, in turn, aggravates oxidative stress, resulting in severe hepatic injury. Although several antioxidant therapies using N-acetylcysteine (NAC), S-adenosyl-L-methionine (SAM), and metadoxine and anti-inflammatory therapies using interleukin 1 (IL1) receptor antagonists, interleukin 22 (IL22) analogue, and anti-TNFα antibodies were reported as promising therapeutic agents for ALD, they showed a short-term improvement even when combined with existing corticosteroids therapy. In this present review, we have highlighted the recent advances in ethanol-induced oxidative stress and therapeutic approaches that affect particularly SOD1 activity in the setting of ALD. Further, we have included the therapeutic potential and molecular mechanism of the nanoformulated SOD1 (NanoSOD) in the amelioration of alcohol-induced liver injury in an experimental model.

## 2. Molecular Mechanism of Ethanol-Induced Oxidative Stress in ALD

Primarily, ethanol is oxidized to acetaldehyde in the cytosol of hepatocytes, catalyzed by alcohol dehydrogenases (ADHs) [15]. Among the six isoenzymes of ADH, ADH1A, ADH1B, and ADH1C are exclusively involved in hepatic ethanol metabolism [16]. Acetaldehyde, a highly toxic metabolite of ethanol metabolism, is converted to a less toxic metabolite, acetone, by aldehyde dehydrogenases 1 and 2 [ALDH1 (cytosolic) and ALDH2 (mitochondrial)]. The acetone is then converted into water and carbon dioxide [17]. Hepatic oxidative stress has been recognized as a characteristic feature of ALD. During excessive ethanol consumption, augmented acetaldehyde reacts with biomolecules including proteins and DNA to generate adducts, resulting in the induction of liver injury. During these enzymatically catalyzed reactions, a more reduced form of nicotinamide adenine dinucleotide (NADH) is produced, causing a reduction in the ratio of NAD+ to NADH (NAD+/NADH) and the generation of ROS such as O_2_•^−^, hydrogen peroxide (H_2_O_2_), and hydroxyl anion (•OH) due to electron leakage from the mitochondrial transport chain [18,19]. The robust generation of highly reactive free radicals and acetaldehyde during ethanol metabolism is facilitated by alcohol ADH and CYP2E1, along with alterations in the antioxidant enzymes including SOD1 and catalase. Of note, ethanol has been reported to attenuate the expression of antioxidant enzymes including SOD1 and deplete glutathione (GSH), a non-enzymatic antioxidant, thereby aggravating oxidative stress in the liver [20,21,22,23].

Long-term heavy alcohol consumption induces the production of more ROS from ethanol oxidation through activation of CYP2E1 [24,25]. Numerous studies have demonstrated the role of CYP2E1 in ethanol-induced oxidative stress and hepatic injury. Over-expression of CYP2E1 in mice and HepG2 hepatocytes potentiates oxidative stress by exacerbating the accumulation of 3-nitrotyrosine (3-NT) and 4-hydroxynonenal (4-HNE) protein adducts [26]. CYP2E1, traditionally known for its microsomal location, is also localized in mitochondria, where it actively metabolizes ethanol. Chronic alcohol consumption induces mitochondrial CYP2E1, which plays an important role in ALD [27]. A study by Bai and Cederbaum evidently demonstrated the central role of mitochondrial CYP2E1 in alcohol-induced oxidative stress and mitochondrial injury with depletion of GSH [28]. Also, mitochondrial CYP2E1 augmented the levels of ROS, 3-NT, and 4-HNE protein adducts and diminished mitochondrial membrane potential [28]. In addition, Cyp2e1-null mice exhibited a decrease in oxidative stress and were protected from ethanol-induced injury [29]. Similarly, administration of Chlormethiazole, a pharmacological inhibitor of CYP2E1, diminished liver injury in chronic ethanol-fed mice [30]. Further, treatment with chlormethiazole in ethanol-fed mice reduced ethanol-induced oxidative stress [29].

Ethanol exposure induces the expression of NADPH oxidase (NOX), a key enzymatic source of ROS, which facilitates the generation of O_2_•^−^ from molecular oxygen using NADH/NADPH as electron donors [31,32,33]. Among NOX enzymes, NOX1 and NOX4 are highly present in the hepatocytes and liver [33,34]. ROS thus produced from NOX interacts with macromolecules including DNA, proteins, and lipids, resulting in the impairment of many cellular functions. It has been reported that CYP2E1-induced ethanol metabolism is directly associated with upregulation of ROS production and NOX4 expression [35,36]. Sun et al. showed that long-term ethanol exposure resulted in overexpression of NOX4 in the hepatic mitochondria. Chronic alcohol consumption increased NOX4 expression in the mitochondrial fraction, whereas knockdown of NOX4 was found to have higher mitochondrial membrane permeability transition (MPT) potential with diminished mitochondrial O_2_•, decreased apoptosis and hepatic steatosis upon exposure to ethanol [32]. NOX has been reported to have a key role in the generation of oxidants in activated macrophages as well [13]. Chronic ethanol-induced ROS generation in Kupffer cells, a hepatic resident macrophage, is dependent on the interaction of NOX and p47phox [37]. Also, NOX-induced production of ROS plays a role in the activation of nuclear factor-kappa B (NF-κβ) and production of tumor necrosis factor α (TNFα) in hepatic Kupffer cells after ethanol treatment [37].

Superoxide dismutase is the principal enzyme involved in the neutralization of O_2_•^−^ into less toxic H_2_O_2_ and O_2_. By doing so, it plays a frontline role in protecting cells from oxidative damage. Hydrogen peroxide is further converted into water by GSH which works synergistically with SOD to offset ROS production. The reactions between GSH and ROS produce glutathione free radical (GS•). This GS• reacts with GSH to form free radical of glutathione disulphide (GSSG•). The GSSG• donates an electron to molecular oxygen, regenerating O_2_•^−^, which is subsequently neutralized again by SOD [38,39]. Excessive production of ROS causes an alteration in the functions of GSH and SOD, leading to progressive depletion of antioxidants and impairment of mitochondrial respiratory chain. This will lead to an irregular mitochondrial shape, reduced mitochondrial protein synthesis with a change in ATP production [40,41,42].

The generation of O_2_•^−^ leads to oxidative stress via different mechanisms. It promotes the formation of lipid peroxidation products, such as malondialdehyde (MDA) and 4-hydroxynonenal (4-HNE), which react with DNA to generate DNA adducts that form outwards-oriented DNA loops. As evidenced, MDA reacts with nucleic acid such as deoxyguanosine, while 4-HNE reacts with deoxyadenosine and deoxycytidine [38,43]. Furthermore, the H_2_O_2_ thus generated from SOD is enzymatically converted into H_2_O through the action of several enzymes, including catalase (CAT), glutathione peroxidases (GPx), and thioredoxin (Trx)-dependent peroxiredoxin (Prx). Alternatively, H_2_O_2_ produces the hydroxyl anion (•OH) through the Fenton reaction in the presence of Fe^2+^. Also, O_2_•^−^ reacts with nitric oxide radicals (•NO) to generate oxidants and nitrating agents such as peroxynitrite (ONOO−) [44]. These highly reactive and toxic radicals can contribute to the development of oxidative stress.

## 3. Cellular and Subcellular Localization of Mammalian SOD Isoforms

Superoxide dismutases (SODs) are ubiquitous metalloenzymes present in eukaryotes and prokaryotes. SODs are characterized as the primary antioxidant defense against ROS. SODs comprise three types in mammals which differ in their location and metalloenzymes. They are as follows: (i) SOD1, also known as Cu/Zn-SOD, is a Cu/Zn-containing metalloenzyme exclusively expressed in the cytosol and mitochondrial intermembrane space; (ii) SOD2, or Mn-SOD, is ubiquitously present in the matrix of the mitochondria; and (iii) SOD3 (Cu/Zn-SOD) exists in the extracellular compartment [45,46]. The three types of SODs are involved in the dismutation of •O_2_^−^ into H_2_O_2_ in the cells. Generally, SOD catalyzes the conversion of •O_2_^−^ produced from many cellular processes into H_2_O_2_ [44].

### 3.1. Functional Role of SOD1

Superoxide dismutase 1 (SOD1) is a free radical scavenger and the coding gene of the human SOD1 gene is 9307 bp and 21q22.11 (gene locus) (Entrez Gene ID 6647). The mature and functional human SOD1 is present as a homodimeric metalloprotein of 32 kDa with two equal, non-covalently connected subunits [47].

The most extensively investigated relationship between SOD1 and human studies is amyotrophic lateral sclerosis (ALS), which is a late-onset severe motor-neuron degenerative disorder [48]. A study by Rosen et al. first identified that a cause of familial ALS is due to a mutation in the SOD1 gene [49]. The estimated cases of SOD1 gene mutations are around 12–20% of people with familial ALS, while they are about 1–2% of people with sporadic ALS [50]. In addition, Zhang et al. reported that patients with the SOD1-251A/G polymorphism have an increased risk of age-related cataracts, owing to their decreased O_2_•^−^ free radical scavengers, including SOD1, CAT, and GPX in eye lenses [51]. Studies using genetically engineered mouse models have elucidated the functional role of SOD1 in modulating the pathogenesis of several diseases. For example, SOD1 knockout (SOD1^(−/−)^) mice showed a higher propensity for hyperoxia and paraquat toxicity [52]. Also, mice knocked out of SOD1 showed severe motor neuron defects after axonal injury [53]. A distinct phenotype was documented in the SOD1^(−/−)^ female mice. Homozygous SOD1^(−/−)^ female mice exhibited a marked decrease in fertility owing to a rise in embryonic lethality compared to heterozygous SOD1-null mice (SOD1^(+/−)^). It is suggested that oxygen free radicals are causing abnormalities in the female reproductive system [52]. Of note, mice ablated with SOD1 were shown to have a decreased survival rate with increased neoplastic alterations in the liver, and about 70% of these SOD1^(−/−)^ mice developed hepatocellular carcinoma (HCC) [54]. Aging is characterized by progressive and irreversible alterations in oxidative stress, overt inflammation, and organ dysfunction. SOD1^(−/−)^ mice display increased age-linked pathologies and tissue injury due to oxidative stress-induced upregulation of p53 [55]. A study by Deepa et al. on frailty, one of the geriatric syndromes, demonstrated weight loss, augmented weakness, low physical activity, and fatigue with a simultaneous increase in inflammation and sarcopenia in SOD^(−/−)^ mice [56]. Similarly, SOD^(−/−)^ mice showed muscle atrophy with a loss of muscle mass and function [57]. SOD1^(−/−)^ mice had age-related cochlear impairment with hearing loss and degeneration of spiral ganglion cells [58]. These studies clearly suggested that defects in SOD1 leads to a number of pathologies.

### 3.2. Functional Roles of SOD2 and SOD3

Superoxide dismutase 2 heterozygous knockout (SOD2^(+/−)^) mice exhibit numerous pathological phenotypes, including a higher propensity to oxidative injury, dilated cardiomyopathy, severe anemia, neurodegeneration, impaired mitochondrial oxidative phosphorylation, initiation of early apoptosis, and poor survival [59,60,61,62,63]. Due to upregulated mitochondrial oxidative damage, homozygous mice deficient in SOD2 had a 100% incidence of cancer during aging [59,64]. Notably, conditional deletion of SOD2^−/−^ in mice, particularly in the brain, was more harmful and caused perinatal death with a progressive neurological disorder, signifying increased susceptibility to neurodegeneration due to exacerbated mitochondrial free radicals [65]. Apart from this, many studies have shown that overexpression of SOD2 is associated with decreased levels of O_2_•^−^ in the mitochondria, inhibition of age-associated impairment in oxidative stress, and retrieval of normal mitochondrial function during aging [66,67,68]. In contrast with SOD1, there was no evidence of age-associated development of cataracts in hemizygous SOD2-null mice [69].

Superoxide dismutase 3 (SOD3), an extracellular SOD, is highly expressed in the blood vessels and lungs and is known for its role in the regulation of vascular functions [70]. SOD3 heterozygous knockout (SOD3^(+/−)^) mice exhibited an aggravated angiotensin-stimulated hypertension due to reduced bioavailability of nitric oxide (NO) in the vascular wall [71]. In addition, SOD3 activity decreased in patients with cardiovascular disease [72]. Generally, under normal conditions, the SOD3 mutant mice develop normally and remain healthy until adulthood. On the other hand, mice deficient in SOD3 under hyperoxic conditions (>99% oxygen) exhibited shorter survival and progressed to an earlier onset of lung edema [73]. Short-term deletion of the SOD3 gene in adult mice by the Cre-Lox method caused severe acute pulmonary injury and mice survived only for a few days after deletion of the SOD3 gene [74]. Thus, each of the SOD isoforms have different roles in regulating the physiological and pathological processes.

## 4. Role of SOD1 on Alcohol-Induced Oxidative Stress in Cell Models

A strong link exists between SOD1 and ALD. However, such an association is not quite evident for SOD2 or SOD3. Although SOD2 is critical for scavenging •O_2_^−^ in mitochondria, evidence suggests that its overexpression worsens mitochondrial DNA depletion after prolonged alcohol consumption in mice [75]. Regarding SOD3, its role in altering ALD remains unclear. Therefore, this review focuses on the role of SOD1 in modulating the progression of ALD. As mentioned, ethanol-induced stimulation of CYP2E1 is a key mechanism contributing to O_2_•^−^ generation in the liver. Human cell line VL-17A (HepG2 cells over-expressing CYP2E1 and ADH) exposed to 100mM ethanol significantly increased oxidative damage via increasing peroxide generation. This was associated with a significant reduction in SOD1 level [76]. Human liver cells (L02) treated with (150 mmol/L) for 48 h exhibited an increased level of MDA with a significant reduction in the levels of antioxidants such as SOD1, GPX and CAT [77]. Also, arachidonic acid and Fe^2+^-induced ROS, lipid peroxidation, and apoptosis in E47 hepatocytes were inhibited by adenoviral-mediated over-expression of SOD1. SOD1 prevented E47 cells against free radical adducts produced from oxidative stress [78]. These studies suggest that ethanol reduces SOD1 in cultured hepatocytes and over-expression of SOD1 is effective in attenuating ethanol-induced oxidative stress.

## 5. Impact of Alcohol Administration Patterns on Hepatic SOD1 and Oxidative Stress in Rodent/Clinical Studies

### 5.1. Chronic Alcohol Feeding

Though the antioxidant enzymes are abundantly found in the liver, the levels of SOD1 and GSH were reduced in rats after chronic ethanol administration [79,80]. Chronic alcohol feeding using the Lieber–DeCarli diet (containing 5% ethanol) for 28 days, following 10 weeks of high-fat diet in C57BL/6J mice, resulted in attenuated hepatic SOD1 activity, with a concomitant upregulation of CYP2E1 expression [22]. C57BL/6J mice fed on chronic ethanol for 32 days exhibited reduced hepatic SOD1 protein levels, along with decreased activity of other antioxidant enzymes including CAT and GPX 1/2, while CYP2E1 expression remained upregulated [81].

In another study, ICR mice administered continuous gastric intubations of 14.2 mL/kg body weight (56% alcohol) for 30 days displayed a reduction in SOD1 levels with a decline of other antioxidant enzymes such as GPX3, CAT, and SOD2 in the hepatocytes [77]. Also, significantly lower gene and protein levels of SOD1 were observed in the hepatic tissue of Male Kunming mice that received chronic alcohol feeding (50% alcohol (*v*/*v*) daily at 0.1 mL/10 g body weight) for 56 days. In addition, the gene and protein expression of other antioxidant enzymes such as SOD2, CAT, and inducible nitric oxide synthase (iNOS) were decreased in the liver of these mice while a marked increase was observed in the serum levels of inflammatory mediators such as IL6, IL12, TNFα and interferon gamma (IFN-γ) [82]. Ethanol reduces the level of SOD1 in rats as well. For example, male Wister rats fed ethanol with corn oil and fish oil showed a reduction in the level of SOD1 along with other antioxidant enzymes, including GPX and catalase. Particularly, rats fed ethanol and fish oil showed a severe liver injury, including hepatic steatosis, necrosis, and inflammation with higher levels of lipid peroxidation. A reduction in antioxidant enzymes in rats fed an ethanol diet with corn oil or fish oil exacerbates the oxidative damage, thereby contributing to an enhanced ALD [80]. Moreover, intragastric administration of ethanol (4 g/kg/day) to the Sprague-Dawley rats for 30 days was observed to reduce SOD1 activity in the liver with other oxidative markers including MDA, GSH, and catalase while increasing ethanol-metabolizing enzymes such as CYP2E1 and ADH [83].

In contrast to previous studies, chronic administration of 30% ethanol (7 g per kg body weight) with or without methamphetamine to male Wister rats for 30 days showed a significantly increased protein level of SOD1, GSH, and GPX1. Also, co-administration of chronic ethanol and methamphetamine significantly increased the levels of MDA, myeloperoxidase (MPO), IL1β and TNFα and promoted hepatic steatosis, necrosis and fibrosis by modulating AKT/PI3K and mitogen-activated protein kinase (MAPK) signaling in rats [84]. In addition, long-term feeding of ethanol diet for 42 days to C57BL/6 mice promoted hepatic steatosis, inflammation, and apoptosis with a concomitant upregulation of hepatic SOD1 and GPX [85]. Watson et al. have shown that WT mice fed chronic ethanol for 42 days showed an upregulation of SOD1 along with markers of oxidative stress and inflammation in the liver [86]. Further, sub-chronic administration of heavy alcohol to the Sprague-Dawley rats resulted in an increase in the mRNA levels of SOD1 and γ-glutamyl transferase (GGT) along with an upregulation of CYP2E1, while depleting GSH in the liver [87].

According to these studies, chronic ethanol feeding leads to up- or down-regulation of hepatic SOD1 and both of which are associated with an increase in markers of liver injury. Since increased SOD1 expression is associated with heightened oxidative stress and/or inflammation, its upregulation in some of these studies may represent a counter-regulatory mechanism aimed at mitigating oxidative stress and attenuate the progression of ALD.

### 5.2. Acute Alcohol Feeding

Male Kunming mice fed on ethanol (5.82 g/kg) for 7 days showed a decrease in SOD1 along with SOD2, CAT, and GPX in the liver [88]. Further, these mice showed an increase in oxidative stress and inflammatory response. Key signaling markers, such as Jun N-terminal kinase (JNK) and extracellular signal-regulated kinases (ERK), were significantly upregulated in mice after exposure to acute alcohol [88]. Thus, acute ethanol impairs antioxidant defense, leading to oxidative stress, inflammation, and activation of key signaling pathways, ultimately contributing to liver injury in mice. In another study, acute intragastric administration of ethanol (5 g/kg body weight) followed by a high-fat diet (HFD) (60% kcal) for 3 days led to an aggravated production of ROS by inhibiting SOD1 levels with a concomitant increase in CYP2E1 through upregulation of hypoxia-inducible factor 1-alpha (HIF-1α) [89]. Thus, acute ethanol administration results in a reduction in SOD1 levels.

### 5.3. Binge Alcohol Feeding

Mice administered a single dosage of ethanol produced a significantly elevated level of ROS and MDA, an indicator of lipid peroxidation, while decreasing the activity of antioxidants including SOD, CAT, and GPx in the liver [90]. Ethanol-binge administration of 5 doses of 2 g/kg ethanol once every 12 h to Wister rats induced prooxidant levels by markedly reducing the activity of SOD1, signifying the potential role of SOD1 as an antioxidant defender to balance prooxidants produced by ethanol in the liver [91]. In another study, chronic ethanol binge in mice for 3 days significantly increased the degree of hepatic steatosis with decreased SOD level and other antioxidants including GSH and CAT in liver [92]. This is accompanied by elevated ROS levels, lipid peroxidation, and a marked decline in antioxidant enzyme activity including SOD1, suggesting a crucial role for SOD1 in altering ethanol-induced liver injury. These studies suggest that an ethanol binge leads to a reduction in SOD1 activity.

### 5.4. Clinical Studies

According to National Institute on Alcohol Abuse and Alcoholism (NIAAA), the recommended weekly alcohol intake is between 7 and 14 drinks for men, and between 4 and 7 drinks for women. When the drinking level exceeds the existing allowed limit, it apparently causes alcohol use disorder (AUD). Many biomarkers are being used as diagnostic indicators for AUD, to monitor the risk of alcohol relapse [93]. Recently, a pilot study for alcohol detoxification was conducted in 55 subjects with chronic AUD, and after a month of residential rehabilitation, they showed altered SOD1 and F2-Isoprostanes, along with monocyte chemoattractant protein-1 (MCP1), an inflammatory mediator. During rehabilitation, the percentage of individuals with physiological SOD1 levels rose from 45% at admission to 80% at discharge, and one month post-discharge, SOD1 levels were significantly associated with well-being and a reduced risk of alcohol relapse [93]. SOD1, as a good indicator for prediction of the risk of early alcohol relapse, could be useful for early identification of AUD, establishing a personalized alcohol-related rehabilitation, and monitoring patients with ALD.

Drinking alcohol is more prevalent among HIV-positive individuals [94]. In comparison to normal alcohol consumers, HIV-positive alcohol consumers exhibited a dramatically enhanced level of oxidative stress by overexpressing oxidative DNA damage, augmented GSSH/GSH ratios, and an increased mRNA level of CYP2E1 in monocytes. On the other hand, a significant decrease in the mRNA level of antioxidant enzymes, particularly SOD1, catalase, glutathione S-transferase kappa 1, and Nrf2, were observed in HIV-positive alcohol users [95]. The upregulated oxidative stress mechanism could be due to impaired oxidant and antioxidant signaling to balance cellular redox homeostasis. The changes in the levels of SOD1 and other antioxidants along with markers of oxidative stress and inflammatory response in different animal models and human subjects with ALD are shown in Table 1.

## 6. Role of SOD1 Deletion or Overexpression in Alcohol-Induced Oxidative Stress in Rodents

Studies using the loss-of-function approach have shown an enhanced liver injury in the absence of SOD1. For example, Kessova et al. have shown that mice lacking SOD1 exhibited an enhanced liver injury, compared to WT mice even on an moderate ethanol diet [96]. They showed that moderate ethanol feeding (7–9 g/kg body weight/day) for 3 weeks promoted oxidative stress-mediated liver injury in SOD1^(−/−)^ mice with increased generation of oxidants including peroxynitrite, protein carbonyls, and lipid peroxidation in the liver while decreasing the levels of GSH and SOD2. This also caused a reduction in hepatic ATP content and mitochondrial injury with increased hepatic 3-nitrotyrosine and reactive nitrogen species in SOD1^(−/−)^ mice receiving the ethanol diet. In another study, they demonstrated that administration of ethanol to SOD1^(−/−)^ mice led to the uncoupling of the ADP-ATP exchange ratio and a reduction in mitochondrial membrane potential (Δψ). Also, the activity of adenine nucleotide translocator decreased through upregulation of proapoptotic proteins involved in the permeability transition including cleaved Bax, Bak, and Bcl-xl [97]. These cumulative mitochondrial alterations in the liver led to the development of necrosis and alcohol-associated liver injury. Alcohol is known to modulate the expression of numerous transcription factors and signaling molecules in monocytes and Kupffer cells (liver macrophages). Alcohol-induced increase in the activity of NFκβ and activator protein 1 (AP-1) and induction of key cytokines such as TNFα and IL6 were downregulated by the delivery of Adenovirus-mediated SOD1 (Ad.SOD1) in mice. In addition, Ad.SOD1 also inhibited acute alcohol-induced gene expression of CD14, the endotoxin receptor [98]. However, the role of SOD1 in alcohol-induced hepatic steatosis remains controversial. Uchiyama et al. found that SOD1^(−/−)^ mice developed increased steatosis due to apoB degradation, which impaired lipoprotein secretion [99]. In contrast, an investigation by Curry-McCoy et al. on the role of SOD1 in female SOD1^(−/−)^ mice (B6129s7-sod1^tm1Leb^) fed a chronic ethanol diet showed an increase in oxidative stress, with no indication of alcohol-induced hepatic steatosis [100]. Regardless, these various studies support the critical role of SOD1 in protecting against the progression of ALD.

Experimental approaches to increasing SOD1 levels were in fact effective in attenuating ALD. Ad.SOD1 delivery reversed early ethanol-induced liver injury in rats by reducing free radical adducts and suppressing pro-inflammatory gene expression, including IL1, TNFα, and NFκβ [101]. In another study, administration of Ad.SOD1 to ethanol-fed rats after liver transplantation increased the graft function and survival rate significantly with a concomitant decrease in the release of transaminases (AST and ALT), necrosis, and apoptosis by blunting the production of free radical adducts [102]. However, the use of Ad.SOD1 in clinical settings is limited due to some limitations in this approach. For example, systemic delivery of adenovirus-mediated drugs leads to accumulation in the liver, which eventually causes hepatotoxicity. In addition, people who have adenoviral neutralizing antibodies will fail to have efficient gene expression [103].

## 7. Available Treatment Options for ALD

### 7.1. Current Therapies for ALD

Alcohol abstinence is the most frequently prescribed preventive and treatment modality for patients with ALD. In patients with early-stage ALD, abstinence can ameliorate hepatic steatosis and liver injury. Despite sobriety, cirrhosis can still be observed in a few people with ALD [104,105]. Liver transplantation is still considered the best treatment for patients with severe AH and non-responders to prednisolone [106]. Though there are no FDA-approved drugs for treating ALD, prednisolone and pentoxifylline have been used for the short-term survival of patients with severe AH. Prednisolone, a corticosteroid-like drug, has been a first-line drug used to treat patients with AH for many years. Prednisolone was reported to attenuate the release of TNFα, while increasing the production of IL10 in patients with AH, increasing the life span, and delaying the progression of encephalopathy [107]. Prednisolone was found to improve short-term survival of patients with AH at an early stage [6]. However, these corticosteroid classes of drugs cannot be used for the long-term survival of patients with severe AH [108,109]. Pentoxifylline is another kind of antioxidant with anti-inflammatory properties for severe AH patients. Similar to prednisolone, therapeutic intervention with pentoxifylline had no significant long-term survival improvement in severe AH patients. Also, pentoxifylline did not show noteworthy survival when paired with corticosteroids. Therefore, pentoxifylline is no longer regarded as an effective medication for long-term survival of people with severe AH [6]. Though these drugs are used to treat people with AUD, they concurrently cause adverse effects such as liver toxicity, and their use for ALD patients is also limited [110].

### 7.2. Immunomodulatory Drugs Against ALD in the Clinical Trial

Interleukin 1 (IL1), a proinflammatory cytokine, acts through the IL1 receptor type 1. An elevated level of IL1 was observed in patients with chronic ALD [111]. Therefore, finding a medication that inhibits the activation of the IL1 receptor has been recommended as an alternative therapeutic approach for patients with ALD. Anakinra is an IL1 receptor antagonist approved by the FDA for rheumatoid arthritis, Still’s disease, auto-inflammatory disease, and various chronic ailments [112,113,114]. Administration of anakinra was found to inhibit inflammasome-mediated alcoholic steatohepatitis in mice [115]. A recent clinical study by Dasarathy et al. demonstrated that a combination of anakinra with other drugs, particularly pentoxifylline and zinc sulfate, led to better clinical results in patients with severe AH when compared with corticosteroid class of drugs [116]. Interleukin 22 (IL22) is reported to have antioxidant, antisteatotic, antiapoptotic, and antifibrotic properties and is a potential candidate for treatment of ALD [117]. Treatment of healthy volunteers with F-625, a human recombinant IL22 molecule, was demonstrated to be safe and tolerable [118]. A recent clinical study also confirmed the safety and effectiveness of F-652 by attenuating key markers of inflammatory mediators, while upregulating markers involved in hepatic regeneration [119]. Another interesting target is TNFα, a proinflammatory cytokine that plays a key role in the development of ALD. TNFα has a key role in the portal and systemic hemodynamic derangements in AH [120]. In patients with severe AH, administration of Infliximab, a monoclonal anti-TNFα antibody, was found to be well-tolerated and clinically safe but did not improve treatment outcome [121,122]. Although IL1 receptor blockade, recombinant IL22 protein, and anti-TNFα antibody were experimentally evaluated as tolerable and clinically safe, these drugs were not as effective in managing ALD.

### 7.3. Antioxidant Therapies for ALD

N-acetylcysteine (NAC) is a well-known antioxidant and GSH precursor. Clinically, NAC is used as a therapeutic agent for the treatment of acute liver failure induced by acetaminophen [123,124]. As mentioned earlier, ethanol-induced oxidative stress plays a major role in the development of ALD. Similar to pentoxifylline and corticosteroids, NAC was also studied as a treatment for ALD. But treatment with NAC alone showed no short-term survival in comparison to corticosteroids or pentoxifylline alone. On the other hand, NAC when combined with prednisolone synergistically showed a marked increase in survival rate; however, no apparent long-term survival effect was observed with NAC treatment [125,126].

SAM is a chief methyl donor produced by methionine adenosyltransferase α1. Chronic ethanol administration significantly reduced the level of SAM in the liver, resulting in progressive alcoholic steatohepatitis. The decreased SAM was found to alter methionine metabolism by affecting DNA and histone methylation in the liver [127]. On the other hand, supplementation of SAM dramatically protected liver and mitochondrial function against ALD, by restoring GSH and attenuating inflammation by downregulating gene expression of TNFα and the upregulation of IL10 synthesis [128]. Mato et al. suggested chronic treatment with SAM would improve long-term survival, delaying liver transplantation in patients with ALD, particularly alcoholic liver cirrhosis [129]. However, a randomized control study by Medici et al. suggested that treatment with SAM for 24 weeks showed no significant survival effect in patients with ALD [130].

Metadoxine, an ionic complex of the pyrrolidone carboxylate and pyridoxine molecule, is another antioxidant agent reported to have a hepatoprotective role against alcohol-induced oxidative stress [131,132]. Metadoxine attenuated redox imbalance in HepG2 hepatocytes and prevented ethanol-triggered TNFα secretion in hepatic stellate cells [132]. It improved liver functions following chronic alcoholism [133]. In patients with severe AH, metadoxine combined with glucocorticoids markedly increased short-term survival and prevented encephalopathy and hepatorenal syndrome [134]. Compared to pentoxifylline alone, metadoxine plus pentoxifylline increased the 3- and 6-month survival in patients with severe AH [135]. Therefore, the combination of antioxidants with corticosteroids or pentoxifylline may be considered as an effective therapy against ALD.

### 7.4. Effects of Nutraceuticals in Altering SOD1 in ALD

Geniposide from *Gardenia jasminoides* Ellis was reported to protect against acute alcohol-induced liver injury in mice through upregulation of the chief antioxidant enzymes. Gene expression as well as enzymatic activity of SOD1 were significantly increased after geniposide administration. Ethanol treatment resulted in the downregulation of hepatic antioxidant enzymes, including GSH, GST, GPx, and CAT, which was prevented by geniposide treatmen [136]. Oleanolic acid (OA), a triterpenoid, has been shown to counteract ethanol-induced oxidative stress by restoring hepatic levels of SOD1 in rats subjected to chronic alcohol exposure. Co-administration of OA with chronic ethanol significantly reduced MDA, GSH and catalase. OA protected ethanol-mediated liver injury by inhibiting the expression of CYP2E1 and ADH and decreasing the inflammatory cytokines including TNFα and IL6 through upregulation of Nrf2 activity [83]. Saponins isolated from *Panax japonicus* were demonstrated to protect against chronic alcohol-induced liver damage in ICR mice by accelerating the expression of SOD1 and other antioxidant enzymes such as glutathione peroxidase 3 (GPX3) and SOD3 [77]. Administration of polyphenols extracted from Camellia sinensis has been shown to upregulate the mRNA and protein levels of SOD1, as well as SOD2, CAT, and nitric oxide synthases (nNOS and eNOS), while downregulating iNOS expression and other inflammatory mediators, including IL-6, IL12, TNFα, and IFNγ, in mice exposed to chronic ethanol [82]. Taken together, these studies show that natural products with liver protective effects against ALD via increasing the expression of SOD1 are useful for alcohol-induced oxidative stress and liver injury. These preclinical studies provide compelling evidence for the effect of the nutraceutical components in ameliorating ALD. However, further clinical studies in human subjects are needed to assess their role as a therapeutic option for ALD.

### 7.5. Effects of Other Drugs in Altering SOD1 in ALD

Recently, Hu et al. demonstrated that Vitamin D deficiency aggravates hepatic oxidative stress and inflammation in mice with ALD. Additionally, vitamin D deficiency downregulated hepatic antioxidant genes, including SOD1 and GPX, promoted glutathione (GSH) depletion and lipid peroxidation in the liver, and exacerbated markers of apoptosis in ALD [85]. These findings underscore a strong association between vitamin D status and hepatic antioxidant enzyme activity, particularly SOD1, in the progression of ALD. Carvedilol, a β-blocker commonly used to treat cardiovascular disease, inhibited the expression of pro-inflammatory factors such as IL1β and TNFα, as well as reduced hepatic ROS levels in chronic alcohol-fed rats. These effects were mediated, in part, by modulating the activation of Kupffer cells and hepatic stellate cells (HSCs). In addition, carvedilol upregulated the expression of antioxidant enzymes SOD1 and GPx1, as well as SOCS1, while downregulating other inflammatory mediators including cyclooxygenase-2 (COX-2), RANKL/RANK, IBA-1, and ICAM-1. It also reversed ethanol-induced hepatosteatosis and fibrosis, thereby preserving liver architecture [137]. Similarly, Roxadustat, a HIF-1α activator used in the treatment of anemia and chronic kidney disease, alleviates ethanol-induced oxidative stress in mice with both chronic and acute alcohol-induced liver disease by attenuating CYP2E1 activity and enhancing hepatic SOD1 expression [89]. However, further studies on the effectiveness of the nutraceuticals or these drugs are still warranted for the long-term survival of patients with ALD.

### 7.6. Effects of Probiotics in Altering SOD1 in ALD

Molecules derived from the gut have been suggested to play a critical role in hepatic oxidative stress and inflammation [138,139,140]. Altering the gut microbiota has been studied as a potential means to alleviate ALD. For example, the therapeutic potential of probiotic treatment in ALD has been investigated in experimental animal and clinical models. *Lactobacillus plantarum* C88 was found to protect against long-term alcohol-induced liver injury in mice by decreasing MDA levels and increasing hepatic SOD activity. These effects were closely associated with downregulation of CYP2E1 and upregulation of *Nrf2* in the liver [140]. *Lactobacillus plantarum* HFY09, another strain, was shown to protect against ethanol-induced hepatic oxidative stress by upregulating the mRNA expression of *Sod1* and other antioxidant enzymes, including *Sod2*, *Gpx*, *Cat*, and peroxisome proliferator-activated receptor alpha (*Ppara*), while downregulating the expression of *Cox1*, *Jnk*, and *Erk* in mice fed alcohol for 7 days [88]. Likewise, *Lactobacillus rhamnosus* GG and *Lactobacillus casei* were reported to have similar antioxidant and anti-inflammatory effects in mice exhibiting alcohol-associated liver injury [138,139]. A similar hepatoprotective effect was observed in Kunming mice fed subacute ethanol and orally administered *Lactiplantibacillus pentosus* CQZC01. *L. pentosus* CQZC01 significantly increased the levels of antioxidant enzymes, including SOD1, SOD2, GPX, and CAT. It also elevated levels of the anti-inflammatory cytokine IL10 and reduced the expression of pro-inflammatory markers such as IL1β, IL6, and TNFα [141]. In another study, administration of *Levilactobacillus brevis* MG5311 to C57BL/6 mice alleviated chronic ethanol-induced liver injury by attenuating hepatic oxidative stress, primarily through upregulation of SOD1 protein levels, along with other antioxidant enzymes such as CAT and GPx1/2 in the liver [81]. Additionally, *Levilactobacillus brevis* MG5311 upregulated key regulators of lipid metabolism, including SIRT1 and PPARα, while attenuating the expression of CYP2E1 and SREBP-1c in the liver, thereby alleviating chronic alcohol-induced liver injury [81]. Hence, supplementation of probiotic candidates such as *Lactobacillus plantarum* HFY09, *Lactiplantibacillus pentosus* CQZC01, and *Levilactobacillus brevis* MG5311 may be useful for the management of ALD via increasing SOD1 in liver. These studies suggest that probiotics could be considered complementary to standard treatment in human patients with ALD.

### 7.7. NanoSOD as a Novel Antioxidant Therapeutic for ALD

Owing to their exclusive function as an effective antioxidant, supplementation of SODs in the form of natural sources has been suggested as useful against various maladies [142]. Although SOD supplementation may be more beneficial due to its various pharmacological properties in comparison to known antioxidants, the pharmacological use of exogenous SODs is limited in clinical practice. Also, oral supplementation is limited because of its low bioavailability due to its high molecular weight, pH, reduced absorption by cells, and proneness to degradation by digestive enzymes [143]. As compared to natural SODs, SOD mimetics are found to have more advantages, such as low molecular weight, altered pharmacokinetics and pharmacodynamics, reduced antigenicity, and increased stability with a longer half-life [144]. Although the SOD mimetics have been studied as an adjuvant therapy to attenuate radiation-induced damage in cancer patients, their effectiveness in treating alcohol-related pathologies remains questionable.

Several studies have focused on the packaging of SODs with suitable delivery systems using nanoparticles to increase their efficacy and half-life. SOD1 has been nanoformulated in a variety of ways, and their effectiveness in treating various ailments has been investigated for more than a decade. Pluronic-modified SOD1 was demonstrated to decrease oxidative stress in cultured neuron cells without inducing toxicity [145]. Manickam et al. synthesized NanoSOD by encapsulating SOD1 in poly-L-lysine (PLL50) and polyethylene glycol (PEG) polymer. The PLL-PEG polymer, in turn, is cross-linked using 3,3′-dithiobis (sulfosuccinimidyl propionate (DTSSP)), a reducible cross-linker, to improve the stability [146]. NanoSOD markedly attenuated oxidative stress by scavenging O_2_•^−^ in cultured brain microvascular endothelial cells and central neurons [146]. In another in vivo study, NanoSOD improved angiotensin II-stimulated hypertension [147]. Findings from our lab further showed the effect of this NanoSOD in ameliorating obesity-associated adipose tissue inflammation [148]. We also showed that NanoSOD significantly improved vascular and aortic inflammation in high-fat diet-induced obesity [149]. In addition, we reported the effect of NanoSOD in reducing the expression of CYP2E1 in mice exposed to a high-fat diet followed by chronic ethanol feeding [22]. Moreover, NanoSOD administration increased the protein level of hepatic SOD1 with a concomitant decrease in markers of liver injury [22]. In a separate study, we showed that NanoSOD treatment decreased hepatic triglyceride (TG) and steatohepatitis caused by chronic ethanol feeding in mice [150]. In this study, NanoSOD showed a 2-fold reduction in the level of CD68 and correspondingly decreased levels of *Ccl2* and *Mmp12* mRNAs, compared to ethanol-fed mice. In addition, significantly increased levels of phosphorylated AMP-activated protein kinase (AMPK) were observed in the livers of ethanol + NanoSOD-administered mice compared to both control and ethanol-fed mice [150]. The chronic ethanol-induced increase in hepatic and/or plasma MCP-1 and C-C chemokine receptor type 2 (CCR2) levels were blunted after NanoSOD administration. In the adipose tissue, NanoSOD increased markers of anti-inflammatory macrophages, in particular, arginase 1 (*Arg1*) in chronic ethanol-fed mice. Ethanol exerts direct effects in adipose tissue, leading to lipolysis and release of free fatty acids. Upregulated CYP2E1 activity is associated with an increase in adipose tissue lipolysis. In the high-fat+ethanol feeding study, we reported that administration of NanoSOD markedly reduced the levels of CYP2E1 in adipose tissue while also increasing adipose tissue mass and decreasing plasma fatty acids [22]. These studies suggest that administration of SOD1 nanoparticles exerts several beneficial effects in the liver and adipose tissue upon ethanol feeding. Thus, therapy targeted towards increasing SOD1 activity may be beneficial in protecting against alcohol-associated organ injury. Taken together, these studies show that various therapeutic agents including nutraceuticals, probiotics, and other experimental drugs, in particular, NanoSOD, attenuate ALD at least, in part, via increasing SOD1 levels and/or activity (Table 2).

## 8. Summary and Future Perspectives in the Development of NanoSOD

**Figure 1:**
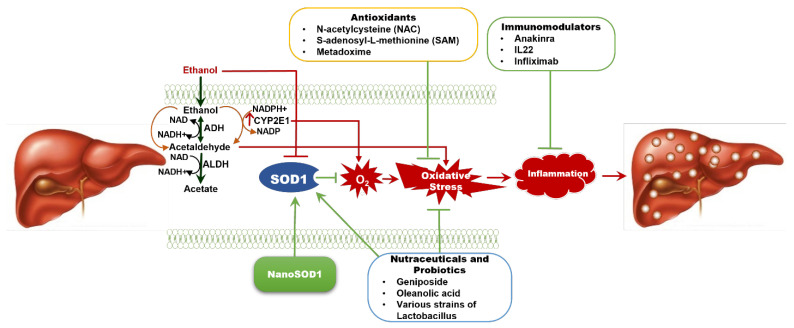
Overview of the Current Therapies for Alcohol-Associated Liver Disease (ALD) and Potential Therapeutic Effects of SOD1. ALD results from chronic alcohol consumption, leading to oxidative stress and a spectrum of liver injury, from simple steatosis to severe fibrosis and cirrhosis. Superoxide dismutase 1 (SOD1) plays a key role in scavenging superoxides and reducing oxidative stress, but alcohol metabolism reduces SOD1 levels in the liver, worsening liver damage. Overexpressing SOD1 has been shown to reverse early liver inflammation. Current therapies for ALD have limited long-term efficacy, but antioxidant treatments like NAC and SAM offer some benefit. Nutraceuticals and probiotics may enhance SOD1 activity and inhibit oxidative stress, thereby attenuating ALD. The therapeutic efficacy of anti-inflammatory agents such as anakinra (an interleukin-1 receptor antagonist) and IL-22 analogues against ALD are under investigation. NanoSOD1, a nanoformulated SOD1, is emerging as a promising therapy to combat oxidative stress and alcohol-induced inflammation, providing potential liver protection in ALD. ADH—alcohol dehydrogenase; ALDH—aldehyde dehydrogenase; IL22—interleukin 22; NAC—N-acetylcysteine; NAD—Nicotinamide adenine dinucleotide; SAM—S-adenosyl-L-methionine.

In summary (Figure 1), ethanol metabolism via alcohol dehydrogenase and CYP2E1 leads to oxidative stress which, in turn, results in inflammation and liver injury. Several lines of evidence suggest that ethanol reduces the SOD1 level/activity in the liver. This can increase O_2_•^−^-mediated oxidative stress, thereby promoting the pathogenesis of ALD. Regarding the treatment options for ALD, all the available drugs have limited long-time efficacy. Due to the strong link between oxidative stress and ALD, several studies investigated the effect of antioxidants in attenuating ALD. Some of them showed a limited benefit in combination with other drugs against ALD. Nutraceuticals and probiotics have been shown to increase SOD1 level/activity which, in turn, can partly mediate their beneficial effects in protecting against ALD in experimental models. Recently, nanoformulated SOD1 has been shown to effectively increase SOD1 level/activity and attenuate ALD in mouse models.

Delivery of SOD1 as a nanoparticle is a promising strategy for treating oxidative stress-related diseases, like cancer, inflammation, and neurodegeneration [151]. NanoSOD can also be combined with other functional nanomaterials for tumor targeting, imaging, and therapy [152]. There are many considerations in developing nanomaterials as a delivery vehicle. Even though NanoSOD is effective against various diseases in experimental models, there are still some challenges and limitations that need to be addressed, such as the evaluation of their biocompatibility and toxicity in vivo, and the elucidation of their pharmacokinetics and biodistribution [153]. The in vivo environment presents distinct hurdles, including heterogeneous microenvironments, variable pH, ionic strength, and the presence of competing biomolecules, all of which substantially impact the activity and stability of nanozymes [154]. Additionally, excessive production and remodeling of the extracellular matrix in cancer and other diseases can greatly hinder nanoparticles from reaching their intended targets, thereby limiting the efficient delivery of therapeutic agents [155]. Moreover, optimizing the properties of nanoparticles to limit unintended immune activation is crucial for overcoming a major barrier to clinical translation. Thus, further research on the design, synthesis, characterization, application, and delivery of NanoSOD is needed to optimize their performance and safety in clinical use.

Since the molecular transport mechanisms differ in humans compared to in vitro and in vivo animal models, the transport properties also change accordingly. The lack of a proper delivery system makes it difficult for NanoSOD to reach the clinical usage. Thus, a new design with more stable and targeted delivery to the liver is needed. A previous report showed that different hydrophilic polymers (cationic, anionic, and neutral polymers) were used to control the surface charges. It is also possible to target a specific tissue by presenting a specific ligand (peptide or antibody) to the polymer [156]. This opens up a new avenue of systemic targeted nanoformulated enzyme therapy for diseases mediated by oxidative stress, in particular, ALD.

## 9. Conclusions

A strong association between ethanol-induced oxidative stress and the pathogenesis of ALD highlights the need for novel antioxidant-based therapeutic strategies. ALD often involves downregulation of SOD1 and many preclinical and clinical drugs have been shown to increase SOD1 level/activity. While current treatments for ALD have limited efficacy, the use of NanoSOD, a nanotechnology-based enzyme therapy, epitomizes a promising new approach to mitigate oxidative stress. Although NanoSOD has effectively attenuated ALD in experimental models, significant hurdles remain for clinical use. These include the need to methodically evaluate its biocompatibility, pharmacokinetics, and biodistribution in humans, and, most importantly, develop a targeted delivery system that can improve the efficacy and reduce the effective dosage. Therefore, continued research on NanoSOD is essential to optimize its design and targeted delivery to make it a safe and effective treatment for ALD.

## Figures and Tables

**Table 1:** Levels of SOD1 and other proteins regulating oxidative stress and/or inflammatory response in rodents and humans exhibiting ALD.

Type of Studies	Study Details	SOD1 Activity	Other Dysregulations	References
*Chronic Alcohol Feeding*				
C57BL/6J mice	HFD (45%, 10 wk) + Lieber–DeCarli diet (5% ethanol, 28 days)	Decreased	↑ CYP2E1 expression	[73]
C57BL/6J mice	Lieber–DeCarli diet (0.75%–5%, 32 days) + Binge ethanol	Decreased	↑ CYP2E1 expression ↓ CAT and GPX1/2	[74]
ICR mice	56% alcohol (14.2 mL/kg BW, 30 days)	Decreased	*↓ GPX3*, *CAT*, and *SOD2*	[69]
male Kunming mice	50% alcohol (*v*/*v*) (0.1 mL/10 g BW, 56 days)	Decreased	↓ *SOD2*, *CAT*, *iNOS* ↑ IL6, IL12, TNFα, IFN-γ	[75]
male Wister rats	ethanol + corn oil + fish oil	Decreased	↓ GPX, CAT	[72]
Sprague-Dawley rats	ethanol (4 g/kg BW/day, 30 days)	Decreased	↓ MDA, GSH, CAT ↑ CYP2E1, ADH	[76]
male Wister rats	30% ethanol (7 g per kg BW, 30 days)	Increased	↑ GSH, GPX1	[77]
C57BL/6J mice	Lieber–DeCarli diet (4% (*w*/*v*), 42 days)	Increased	↑ *GPX*	[78]
C57BL/6J mice	Lieber-DeCarli ethanol (42 days)	Increased	↑ oxidative stress, inflammation	[79]
Sprague-Dawley rats	heavy alcohol (22% and 38%)	Increased	↑ GGT, *GPX1*, *CYP2E1* ↓ GSH	[80]
*Acute Alcohol Feeding*				
Male Kunming mice	ethanol (5.82 g/kg BW, 7 days)	Decreased	↓ *SOD2*, *CAT*, *GPX* ↑ oxidative stress, inflammation, *JNK*, *ERK*, *COX1*	[81]
WT mice	ethanol + HFD (60%) (5 g/kg BW, 3 days)	Decreased	↑ CYP2E1, HIF-1α	[82]
*Binge Alcohol Feeding*				
Mice	a single dosage of ethanol (3 g/kg BW)	Decreased	↑ ROS, MDA ↓ CAT, GPX	[90]
Wister rats	5 doses of ethanol (2 g/kg BW once every 12 h)	Decreased	↑ prooxidant levels	[91]
C57BL/6 mice	chronic ethanol binge (3 days)	Decreased	↓ GSH, CAT	[92]
*Clinical Studies*				
55 subjects with chronic AUD	residential rehabilitation (30 days)	Increased (during rehabilitation)	Altered F2-Isoprostanes, and MCP1	[86]
HIV-positive Individuals	alcohol consumers	Decreased	↓ *GSSH/GSH ratios*, *CYP2E1* ↓ *CAT*, *GSTK1*, *NRF2*	[88]

ADH—alcohol dehydrogenase; CAT—catalase; COX—Cyclooxygenase; CYP2E1—cytochrome P450 family 2 subfamily E member 1; ERK—extracellular signal-regulated kinase; GGT—gamma-glutamyl transferase; GPX—glutathione peroxidase; GSH—glutathione; GSSH—glutathione disulfide (oxidised); GSTK1—glutathione S-transferase kappa 1; HIF-1α—hypoxia-inducible factor 1-alpha; IFN—interferon; IL—interleukin; iNOS—inducible nitric oxide synthase; JNK—jun N-terminal kinase; MCP1—monocyte chemoattractant protein-1; MDA—malondialdehyde; NRF—nuclear factor erythroid 2-related factor 2; ROS—reactive oxygen species; SOD—superoxide dismutase; TNF—tumor necrosis factor. ↑ = increased (or) upregulated and ↓ = decreased (or) downregulated.

**Table 2:** Role of Nutraceuticals, Other Drugs, and Probiotics in Altering SOD1 Levels.

Types of Therapeutics	SOD1 Activity	Other Dysregulations	References
*Neutraceuticals*			
Geniposide (*Gardenia jasminoides* Ellis)	Upregulated	↑ GSH, GST, GPx, CAT	[123]
Oleanolic acid	Upregulated	↑ Nrf2 ↓ CYP2E1, ADH, TNFα, IL6	[76]
Saponins (*Panax japonicus*)	Upregulated	↑ *GPX3*, *SOD3*	[69]
polyphenols (*Camellia sinensis*)	Upregulated	↑ SOD2, CAT, nNOS and eNOS ↓ iNOS, IL6, IL12, TNFα, and IFNγ	[75]
*Other Drugs*			
Carvedilol (β-blocker to treat CVD)	Upregulated	↑ GPX1, Kupffer cells and hepatic HSCs, SOCS1 ↓ ROS, IL1β, TNFα, COX2, RANKL/RANK, IBA-1, and ICAM-1	[124]
Roxadustat (HIF-1α activator)	Upregulated	↓ CYP2E1	[82]
*Probiotics*			
*Lactobacillus plantarum* C88	Upregulated	↓ CYP2E1, MDA ↑ NRF2	[127]
*Lactobacillus plantarum* HFY09	Upregulated	↑ *SOD2*, *GPX*, *CAT*, and *PPARα* ↓ *COX1*, *JNK*, and *ERK*	[88]
*Lactobacillus rhamnosus* GG and *Lactobacillus casei*	Upregulated	Antioxidant and anti-inflammatory activity	[138,139]
*Lactiplantibacillus pentosus* CQZC01	Upregulated	↑ *SOD2*, *GPX*, *CAT*, IL10 ↓ IL1β, IL6, and TNFα	[141]
*Levilactobacillus brevis* MG5311	Upregulated	↑ CAT, GPx1/2, SIRT1, PPARα ↓ CYP2E1, SREBP-1c	[91]
*NanoSOD*			
NanoSOD1	Upregulated	↓ oxidative stress in Brain, angiotensin II-stimulated hypertension, obesity-associated adipose tissue inflammation, vascular and aortic inflammation, *CYP2E1*, *CD68*, *CCL2* and *MMP12* ↑ *AMPK*, *MCP-1*, *CCR2*, *ARG1*	[73,133,134,135,136,137]

ADH—alcohol dehydrogenase; AMPK—AMP-activated protein kinase; ARG1—arginase 1; CAT—catalase; CCL2—CC motif chemokine ligand 2; CCR2—C-C chemokine receptor type 2; CD68—cluster of differentiation 68; COX—cyclooxygenase; CYP2E1—cytochrome P450 family 2 subfamily E member 1; eNOS—endothelial nitric oxide synthase; ERK—extracellular signal-regulated kinase; GPX—glutathione peroxidase; GSH—glutathione; HSCs—hepatic stellate cells; IBA-1—short for ionized calcium binding adaptor molecule 1; ICAM-1—intercellular adhesion molecule 1; IFN—interferon; IL—interleukin; iNOS—inducible nitric oxide synthase; JNK—jun N-terminal kinase; MCP-1—monocyte chemoattractant protein-1; MDA—malondialdehyde; MMP12—matrix metalloproteinase-12; nNOS—neuronal nitric oxide synthase; NRF—nuclear factor erythroid 2-related factor 2; PPARα—peroxisome proliferator-activated receptor alpha; RANKL/RANK—receptor activator of nuclear factor kappa-B; ROS—reactive oxygen species; SIRT1—Sirtuin 1; SOD—superoxide dismutase; SOCS1—suppressor of cytokine signaling 1; SREBP-1c—sterol regulatory element-binding protein 1c; TNF—tumor necrosis factor. ↑ = increased (or) upregulated and ↓ = decreased (or) downregulated.

## Data Availability

No new data were created or analyzed in this study. Data sharing is not applicable to this article.
